# Parents’ concerns about future pregnancy after stillbirth: a qualitative study

**DOI:** 10.1111/hex.12480

**Published:** 2016-08-01

**Authors:** Sarah Meaney, Claire M. Everard, Stephen Gallagher, Keelin O'Donoghue

**Affiliations:** ^1^ National Perinatal Epidemiology Centre University College Cork Cork Ireland; ^2^ Pregnancy Loss Research Group Department of Obstetrics and Gynaecology University College Cork Ireland; ^3^ Cork University Maternity Hospital Cork Ireland; ^4^ Centre for Social Issues Research Department of Psychology University of Limerick Limerick Ireland

**Keywords:** fathers, mothers, qualitative, stillbirth, subsequent pregnancy

## Abstract

**Objectives:**

As stillbirth has a devastating impact, it is imperative to understand the importance of clinical and emotional care after stillbirth and how it influences subsequent pregnancies. The aim of the study was to gain insight into the consideration and planning of a subsequent pregnancy by parents in the weeks following stillbirth.

**Design:**

A qualitative semi‐structured interview format was utilized. Interpretative phenomenological analysis was employed as the analytic strategy.

**Participants and setting:**

The recruitment strategy focused on couples whereby the parents of ten stillborn babies were contacted; however, five men declined to participate in the study. The final sample of 15 parents were all Irish: ten of whom were female and five of whom were male.

**Results:**

Findings revealed two superordinate themes relating to a subsequent pregnancy after stillbirth: aspirations for future pregnancy and expectations of future care. Parents disclosed how the prospect of a subsequent pregnancy was daunting with fears about the potential loss of another child. Despite these fears, parents’ aspirations differed in the days following stillbirth; mothers wished to plan a future pregnancy while fathers were reluctant to consider any pregnancies. Parents were unsure of what to expect in terms of the level of care that would be provided to them in a subsequent pregnancy. Additional appointments at the maternity hospital were considered crucial to provide reassurance during a subsequent pregnancy.

**Conclusions:**

These findings underscore the far‐reaching and contrasting effects of stillbirth on parents. These complex needs highlight the importance of the multidisciplinary team approach.

## 1 Introduction

As a bereavement, stillbirth has considerable impact on parents. Throughout the literature, the definition of stillbirth varies from 20 to 28 weeks of gestation. In Ireland, a stillborn baby is defined as an infant born with no sign of life weighing 500 g or more and/or having a gestational age of 24 weeks or more. Research shows that parents experience distress and sadness as they grieve for their baby and it has been found that how parents are counselled following stillbirth has lifelong impact.^1^, ^2^, ^3^, ^4^, ^5^ From the early literature, it was recognized by the medical community that women who were bereaved by stillbirth would be distressed, but it was felt that it would be in the best interest of the woman to have another baby in order to help put the loss behind her.^1^ In recent years, parents have become more involved in how they are cared for, as they reclaim control over their experiences.^6^ With the recognition of the significance of stillbirth, there has been some progress made^7^, ^8^ including a shift in focus with research examining how best to support and care for bereaved parents.^9^, ^10^, ^11^

The Royal College of Physicians of Ireland (RCPI) state that supportive bereavement care should be offered following stillbirth, which parents may choose to access at their own discretion.^12^ Both the Royal College of Obstetricians and Gynaecologists and the RCPI also recommend that in a pregnancy following stillbirth, women should attend an obstetric consultant‐led antenatal clinic and these women should have early access to care.^12^, ^13^ Despite these developments, there is still no clearly defined pathway of care for these women. Uncertainty surrounding the guidance given to health‐care staff on how best to care for parents following stillbirth^14^ particularly in relation to the next pregnancy remains.

One of the facets of care that remains controversial is related to the timing of a subsequent pregnancy.^15^ Studies have shown that over half of women who have a perinatal loss will become pregnant again,^15^, ^16^ and many will do so within the year.^17^ Research also indicates that women seek medical guidance regarding the optimal time for a subsequent pregnancy following a stillbirth.^15^ Pregnancies following perinatal loss are reported as being anxiety‐laden due to parental fear of recurring loss;^18^ thus, some clinicians recommend that parents need time to recover emotionally before another pregnancy.^16^ Previous studies have reported that there were a number of determinants influencing the timing of the next pregnancy including, but not limited to, advice from relatives, guidance from medical professionals, maternal age, and other social factors.^16^

Given the devastating impact of stillbirth, it is imperative that health‐care professionals understand the importance of clinical and emotional care after stillbirth and how it influences subsequent pregnancies for both women and men. To date, much of the research on stillbirth is focused on the mother's experience; however, studies have shown that perinatal loss can place considerable strain on the relationship between bereaved parents.^1^, ^11^ Therefore, the purpose of this study was to examine both mothers and fathers’ experiences with the aim of gaining more detailed insight into the consideration and planning of a possible future pregnancy by both parents following stillbirth.

## 2 Methods

For the purpose of this study, an interpretative phenomenological analysis (IPA), which has its theoretical foundations in phenomenology, was undertaken.^19^ IPA is increasingly been used in health‐care research as its ideographic approach facilitates researchers to rigorously explore specific psychosocial phenomena which may impact on patient care.^20^ IPA is a qualitative methodology which is focused on the perceptions of individuals as they make sense of a given life‐experience and highlights how this subjective experience is only accessible through interpretation.^20^ Thus, IPA as a method aims to investigate how individuals reflect on and draw meaning from a significant life event. The experience of such an event is often not exclusive to one individual but can be experienced or shared with other individuals, in the case of this study, a pregnancy ending in stillbirth.

### 2.1 Recruitment

This study originated from a study undertaken to examine parental decision making relating to perinatal autopsy; full details of which are reported elsewhere.^24^ During the course of the interviews, aspirations for future pregnancies arose from the participants’ narratives. From these interviews, there was an indication that there were disparities between men and women in relation to pregnancy following stillbirth. In order to fully explore this theme, a second stage of recruitment was undertaken. These additional participants were recruited from a patient list of those who had had a stillbirth at a large tertiary maternity hospital in Ireland. In keeping with the original recruitment process, parents were initially informed of the study by a specialist bereavement and loss midwife. If parents indicated that they were interested in participating, the lead researcher made contact by telephone in order to provide additional information in relation to the study and arrange interviews.

### 2.2 Sample

In maternity‐related research, women often act as gatekeepers for men. Given that this study wanted to examine the experiences of both mothers and fathers, the recruitment strategy focused on couples. Overall, ten couples were contacted, and ten mothers and five fathers agreed to take part. The final sample of fifteen parents were all Irish: ten of whom were female and five of whom were male (Table 1).

**Table 1:** Overview of the sample

Participant	Parent	Baby	Stillbirth type	Gestation	Patient status
Participant 1	Mother	A	Intrapartum	Preterm	Public
Participant 2	Father	B	Intrapartum	Preterm	Private
Participant 3	Mother	B	Intrapartum	Preterm	Private
Participant 4	Mother	C	Antepartum	Preterm	Public
Participant 5	Father	C	Antepartum	Preterm	Public
Participant 6	Mother	D	Antepartum	Very preterm	Public
Participant 7	Father	E	Antepartum	Term	Public
Participant 8	Mother	E	Antepartum	Term	Public
Participant 9	Mother	F	Antepartum	Preterm	Public
Participant 10	Father	F	Antepartum	Preterm	Public
Participant 11	Mother	G	Antepartum	Preterm	Private
Participant 12	Mother	H	Antepartum	Term	Private
Participant 13	Mother	I	Antepartum	Term	Public
Participant 14	Father	I	Antepartum	Term	Public
Participant 15	Mother	J	Antepartum	Preterm	Private

### 2.3 Ethics

Ethical approval for the study was granted by the Clinical Research Ethics Committee of the Cork Teaching Hospitals (Ref ECM 4 (zz) 10/01/12). All participants provided written informed consent.

### 2.4 Data collection

Once recruited to the study parents were interviewed in relation to their experiences of stillbirth. Interviews took place between 4 and 16 months following stillbirth. All semi‐structured interviews were conducted on a one‐to‐one basis in either a room onsite in the hospital or a location convenient to the participant. Interviews were undertaken by SM or, upon request, by SG as two fathers indicated their preference to be interviewed by a male. The semi‐structured interviews were guided by an agreed interview schedule. The interview schedule provided an overview of the areas that the research was concerned with including experience of the pregnancy, expectations of birth, experience of stillbirth, understanding of medical investigations following stillbirth, any considerations for a future pregnancy and finally expectations of future care in the maternity services. Semi‐structured interviews were conducted to allow participants to introduce and discuss areas they consider important which may have not been included in the initial interview schedule. Interviews lasted between 35 and 95 minutes, were digitally recorded and transcribed verbatim.

### 2.5 Data analysis

Transcripts of the interviews were analysed by utilizing an interpretative phenomenological approach.^19^, ^20^, ^21^, ^22^, ^23^ The analysis, as outlined by Smith et al.^19^ was carried out manually. The analysis, which was undertaken by both SM and SG, included the following five stages. Firstly, it involved familiarization whereby each transcript is read and re‐read in conjunction with the corresponding audio recording. Secondly, the researchers independently identified emerging themes from each of the individual transcripts following a line‐by‐line analysis of the texts. Emerging themes are recorded as phrases or sentences throughout each individual transcript which aim to capture the essential qualities of data. As patterns and connections were identified across the transcript, similar themes were grouped together independently by the researchers. Throughout the process, as the researcher interpreted the data, emerging themes were defined and redefined with the integration of cases undertaken collectively by SM and SG. A master list of superordinate and subordinate themes was then created. The final stage was the production of a summary table noting each participant's contribution to the themes and supported by extracts from the transcripts.

## 3 Results

Interviews with the bereaved parents revealed how they experienced disbelief, shock, distress, anger, guilt and sadness as well as reporting how they felt a sense of failure following stillbirth. These emotions are tied up in the parents’ search for meaning as they try to come to terms with what has happened and why it has happened to them, as has been previously reported.^24^ Analysis revealed that as parents begin to grieve their stillborn babies, their thoughts were not solely focused on the baby they had just lost but were also focused on the impact this loss would have on potential future pregnancies.

Two superordinate and six subordinate themes were identified (Table 2). The following two superordinate themes are now discussed relating to potential future pregnancy:

**Table 2:** Superordinate and subordinate themes

Superordinate theme	Subordinate theme
Aspirations for a future pregnancy	Fear of recurrent lossUnhelpful societal responsesConflicting parental aspirationsDisengagement
Expectations of future care	Reassuring medical guidance and supportNeed for consistent specialized care

1. Aspirations for future pregnancy
2. Expectations of future care

### 3.1 Aspirations for future pregnancies

#### 3.1.1 Fear of recurrent loss

Findings indicate that in the days following stillbirth, parents begin to consider the possibility of a future pregnancy. Parents in this study revealed the impact that a stillbirth had on their expectations for a family as their plans were drastically and unexpectedly altered. Following the stillbirth, these parents began to re‐evaluate the process of pregnancy as each of the parents discussed their fears about the potential loss of another baby.

I know that sounds stupid to wonder about stuff in the future but that's the way I look at it like when we got pregnant with baby, we expected to have a baby after nine months and the thing is if she got pregnant again your running the risk of doing the whole thing over again

(Participant 2; Father)

While reflecting on the stillbirth, parents’ previous assumptions about the risk of adverse outcomes in pregnancy were irrevocably altered. When they had embarked on this pregnancy, they had not been fully aware of their risk of stillbirth. They discussed how their expectations rose once they had successfully progressed through the first trimester. Many of the parents spoke about how they considered the pregnancy to be a very positive experience as the mothers “flew” through the pregnancy without incident and how this, at the time, had reassured them. These parents emphasized how they had no indication or sign of what it was they were going to experience. To then have a stillborn baby was a bewildering experience that left these parents questioning what had happened and why it had happened to them. This meant that a number of these parents now adopted a fatalistic approach to pregnancy. They stated that although health‐care staff informed them about their level of risk, they were not reassured and felt it was very likely that they could experience stillbirth again in a future pregnancy.

Yeah and they basically told us that we had the same chance of it happening to us again as if it had never happened to us. The same as any couple walking down the street. But the only thing I kept saying was that it did happen to us you know so they can't give any reassurances.

(Participant 9; mother)

#### 3.1.2 Unhelpful societal responses

Both mothers and fathers disclosed how the prospect of a future pregnancy was daunting, referring in particular to the toll it would take on them mentally. Following the stillbirth, family and friends would try and comfort them by reassuring them with platitudes. They were told that they were young and that they would plenty of opportunities to have more children. These parents remarked about how hurtful those statements were irrespective of their personal intentions to pursue another pregnancy or not. Parents disclosed how difficult and isolating an experience it was. If they were considering a future pregnancy, they found it challenging to openly discuss their concerns as comments from family and friends reinforced the societal belief that having children was just a natural part of life and not the anxiety‐ridden prospect they were now imagining.

If she was to get pregnant and we found out tomorrow, I'd, before she'd get pregnant I'd want to know is there a big, big chance of this happening again or is there, what's the chances [of stillbirth]? And being honest I don't know what the chances [are] so until I know the chances or until I feel someway safe that's not going to happen again. That's something that's always going to be in the back of your head too, even if she was to get pregnant I'd still have it in the back of my head what happened last time round. You know I'd just be a nervous wreck during the pregnancy…one little kick and I'd just be, I don't know, I wouldn't even sleep

(Participant 5; father)

#### 3.1.3 Conflicting parental aspirations

Before the stillbirth, the parents recalled how pregnancy and childbirth were considered a part of life. These parents had hopes and expectations of having a certain number of children in their family and the experience of stillbirth now forced these parents to contemplate and reconsider these life goals. There were disparities observed between the aspirations of the men and women in this study.

Findings revealed that the mothers in this study started planning their next pregnancy in the days following their stillbirth. Despite their fears of another perinatal loss, mothers wanted to proceed with planning a future pregnancy without delay. Many of the mothers spoke of the sense of failure they felt after the stillbirth. They were motivated by parenthood and the implications that their stillborn baby had on their family. There was great importance placed on the status of the stillborn baby within the families, in particular acknowledging their place within the birth order. However, many of the parents had intended on having more children after the pregnancy which ended in stillbirth and mothers in particular wanted to fulfil those aspirations.

my husband was saying he didn't want any more children. So I don't know if you count this much but someone who has lost a baby, for me I would have been pregnant coming out of the hospital again I wanted to be pregnant again that badly and then he was saying he didn't want any more at all and he wouldn't discuss it until after we got the results from the hospital.

(Participant 6; mother)

But she's our fourth but we have three children if you know what I mean…*Husband* is adamant that we have had enough children like Husband would say that we are very blessed…but I think part of it with him is not that he doesn't want any more children, he wouldn't want to have another pregnancy you know because of the outcome with baby

(Participant 9; mother)

The fathers interviewed expressed a clear reluctance to consider any future pregnancies in the months following stillbirth. Some of the men considered the prospect of not having any more children as they perceived the stillbirth as evidence that there was something wrong, they were possibly genetically incompatible with the mothers of their baby. These fathers highlighted how pregnancy was a biological process which they had no control over and they also identified grave concerns for the possible impact of another pregnancy on themselves and their partners, both physically and emotionally. These men recounted how they saw their role as the primary support for their partner through their current loss and any potential future pregnancy. Throughout the interview, the men restated on a number of occasions how they felt they had to be strong emotionally, at times putting aside their own grief, in order to be able to successfully provide such support. This illustrates how these men carry a different burden to the women, thus compounding their fears and reinforcing their reluctance to proceed with another pregnancy.

It's something that's always in the back of your head, and in a way it kind of frightens you to have kids again…because I would be afraid to kind of, I didn't say this her now or anything but I would be afraid to kind of have a baby again in case the same thing would kind of happen. It would kind of frighten you, obviously you don't want to go through that again … I would be dreading it if she was pregnant again…I would be afraid of if it did happen again she would be, the two of us would be devastated. Even worse

(Participant 5; Father)

#### 3.1.4 Disengagement

Communication played a key role in how parents were able to navigate through this experience. Many of these parents, especially the men, spoke about how their concerns about a future pregnancy were often left unspoken. For some of the women in this study, the unwillingness of their partners to consider, or discuss, a future pregnancy had a detrimentally effect on them emotionally. One mother whose husband initially refused to consider a future pregnancy in the first months following their stillbirth likened the experience to suffering another loss. This mother recounted how her husband refused to engage in any discussions about a future pregnancy until they had the results of all the investigations undertaken following the stillbirth. She spoke of feeling anger and resentment during those months especially as she felt her preoccupation with the conflict between her and her husband hindered her ability to grieve the loss of her stillborn baby.

At least I know that door isn't shut because I think that was, that was a second, grief it was sort of like I've lost one baby and I've lost any chance of having another one. So it was like two separate things I was grieving over and I felt guilty because I felt like, half the time I wasn't grieving for Baby I was grieving for the loss of some future baby, instead of thinking about her so once we got past the results I could just concentrate on baby again, as opposed to other stuff.

(Participant 6; mother)

### 3.2 Expectations of future care

#### 3.2.1 Reassuring medical guidance and support

One of the concerns for all the parents when considering a possible pregnancy was what to expect in terms of care in a future pregnancy. The women in this study said that being given practical information on what to expect and what to do in the next pregnancy was most useful. They were appreciative of clear guidance from staff about issues such as hospital appointments and the implementation of possible preventative measures such as taking aspirin or adjusting their diet and exercise routines. This information was generally imparted when they met with a consultant sometime after the stillbirth. The parents noted the importance of the time dedicated to this meeting as it facilitated the opportunity to ask questions about the stillbirth but also future pregnancies. Both doctors and midwives had also given an indication to some parents that they would be monitored more frequently in a future pregnancy following stillbirth. This was a relief to parents because if they chose to become pregnant again, they stated how they would want additional appointments with the maternity hospital for reassurance purposes. The information and guidance provided by staff helped these parents to contemplate a way to navigate through what would be an unknown experience to them, pregnancy after stillbirth. One participant placed such high value on this information that they carried a card with this written detail around with them on their person every day, so that they could implement any recommendations immediately if they were to become pregnant.

She [midwife] gave us a list, I have it in my bag, of everything to do the next time it's like taking aspirin, I'll be brought up for a scan every kind of three to four weeks, you know things like that… she said that I would be closely monitored, it was definitely a big relief for the two of us like when she told us that. You know, so she was very helpful

(Participant 4; mother)

it was hard to come back to the hospital but it was good to get it done [debriefing with the consultant following the stillbirth] …she answered all our questions and she addressed an important thing as well cause we had already decided that we were going to try again to try for another baby at some point so she answered all our questions and I know I'll be looked after very well [in a future pregnancy]

(Participant 3; mother)

#### 3.2.2 Need for consistent specialized care

However, for some parents, they were not given enough specific details about the level of care they would receive. They had concerns that although they would be monitored more closely, they potentially would not be seen as much as they would like.

Obviously you would love that they would have said ‘look you can have more [children] and we found out what was wrong and we now know and that'll never happen’. But obviously that just wasn't the case, she [consultant] did say that if wife ever did get pregnant again that it would be a totally different pregnancy and that she'd be seen all the time. She was been seen every two months or something like that every eight weeks, I don't know. But she said she'd be seen a lot sooner a lot quicker and you know they'd be checking things, scans and stuff, all the time and it'd be a pure, she called it a medical pregnancy…but its words as well…it's easier to say oh we're going to do this we're going to do that but both myself and wife have both said what happens if instead they say right instead of eight weeks we want do it every six weeks or seven weeks it's not really that much more…they're not going to let you come every week or every second week but maybe that's what you would want

(Participant 2: father)

Given these concerns having their care transferred to a “high risk” antenatal clinic in future pregnancy, for some, was important. It would ensure they had access to the individualized and specialist care they felt necessary to proceed with another pregnancy.

Of course you would have concerns, course you would have concerns but em we I suppose to alleviate that we know Wife will receive better care this time because she will be high risk and eh you know you have to trust again or you know you have to get on with things and put your trust in these people who know the most about these things you know. And that we will have a better outcome

(Participant 7; father)

## 4 Discussion

### 4.1 Main findings

The findings from this study indicate that parents immediately reflect upon the possibility of another pregnancy following stillbirth. Two superordinate themes relating to future pregnancy were identified: aspirations for future pregnancy and expectations of future care. The thought of a possible pregnancy was a daunting prospect for both mothers and fathers with fears about the potential loss of another child at the forefront of their minds. Although both parents expressed fear, mothers were driven to plan a future pregnancy while fathers were reluctant to consider any pregnancies. Following stillbirth concerns were raised by parents about the possible emotional impact of another pregnancy. Another concern, shared by both mothers and fathers, was what to expect in terms of the level of care that would be provided in a future pregnancy. Additional appointments at the maternity hospital were considered crucial in order for the parents to be reassured during a subsequent pregnancy.

### 4.2 Strengths and limitations

One of the key elements of qualitative analysis is the identification of any potential factors which may influence the results. This study was undertaken in one maternity hospital which has a dedicated bereavement and loss team. The presence of this specialized team may be influential in two ways. Firstly, the participants were informed about the study by a midwife from the team who was involved in their care. Although the researchers who interviewed the parents are not part of the clinical team, having been recruited via the midwife providing their bereavement support may have influenced their responses. Secondly, not all maternity hospitals have such a dedicated bereavement team. Therefore, the experiences of these parents may differ from those who experience stillbirth in another unit, where staff may not place the same importance on discussing future pregnancy with bereaved parents.

As a methodology IPA acknowledges, the experience of these parents will never be entirely accessible to the researcher and the interpretation of these events is influenced by the researchers own experiences and perspective. As a consequence, the analysis of qualitative data from a differing perspectives may produce different results. Bearing this in mind, data analysis for this study was initially undertaken independently by the authors SM and SG: a female health sociologist and a male health psychologist. The final stages of analysis and their interpretation were then jointly undertaken by SM and SG. The results of these analyses were then presented to CE, a midwife, and KOD, a Consultant Obstetrician, for review. Notwithstanding these limitations, the value of this study is that these findings build on the current body of knowledge by providing additional insight into previously published quantitative findings.

### 4.3 Interpretation

As indicated in this and previous studies, giving birth to a stillborn baby takes an enormous emotional toll on parents.^5^, ^11^ There have been significant changes to the care offered to parents^3^, ^4^, ^10^ as studies have emphasized how the manner in which families are cared for is critical for their ability to cope.^9^, ^10^, ^11^ One of the aspects of care which continues to garner attention is the timing of a subsequent pregnancy. Early literature suggested that mothers should focus on having another baby immediately,^1^ while more recent practice suggests that clinicians recommend parents take the time to recover emotionally before embarking on another pregnancy.^16^ Yet Salfund et al. found that mothers were dissatisfied when advice was given on the timing of a subsequent pregnancy. These mothers stated that the decision of when to conceive again was a matter of personal choice.^15^ Our study echoes these findings as parents stated that when they were counselled following stillbirth, they were most concerned with gauging the risk of recurrent stillbirth if or when they decided to conceive again, as opposed to focusing on the optimal timing of a future pregnancy.

Our study indicated that there was a disparity between mothers and fathers in relation to their aspirations for a future pregnancy. Mothers, in this study, were driven by the desire to have children and indicated that they wished to continue with another pregnancy, although aware that they would be hypervigilant in a future pregnancy. However, fathers expressed a clear reluctance to proceed with another pregnancy. Our study is in keeping with Mills et al.^25^ whose findings indicate that during a subsequent pregnancy, parents suffer similar anxieties and fears and identify how fathers do not openly express these concerns. This is of particular importance as previous studies show that stillbirth has an adverse effect on bereaved parents’ relationships,^1^ with one in eight parents experiencing anxiety or depression.^26^ Badenhorst emphasizes that this is even more the case if parents’ grief is not experienced in tandem.^1^ A study by Lin and Lasker examined the patterns of grief following pregnancy loss, including stillbirth, and found that overall, there was a decline in grief after 2 years.^26^ Although Lin and Lasker reported a normal grief trajectory, from high to low after 2 years, they also identified seven different grief patterns during the 2‐year period.^27^ The findings from Lin and Lasker illustrate the complex and individualistic nature of the grieving process.^27^ Badenhorst further states that health‐care providers should identify specific interventions that will help support both mothers and fathers individually following stillbirth.^1^ Our study further illustrates how both mothers and fathers have potentially different requirements for their follow‐up care after stillbirth. Given the expected grief trajectory of these parents, timely follow‐up care would be beneficial.

All parents in our study revealed a fear of recurrent stillbirth. In a recent review of the risk of recurrent stillbirth, Lamont et al.^28^ stated that unexplained stillbirth is a poorly studied complication of pregnancy and priority must be given to establishing the cause of death in order to counsel parents appropriately about the risk of stillbirth in future pregnancies. Evidence to date suggests that women are at a higher risk of stillbirth in a future pregnancy if their stillbirth was in their first pregnancy.^28^ Clinical management should therefore take into account pregnancy history as well as making use of pre‐pregnancy counselling services following stillbirth.^28^ Such counselling services may be of the utmost importance as the fear of recurrent stillbirth may result in prolonged delays or avoidance of future conception.

## 5 Conclusion

The findings of this study underscore the far‐reaching and contrasting effects of stillbirth on parents. These findings have implications not only for the psychological well‐being of parents but also for clinical practice. The mothers and fathers interviewed illustrated differing needs and concerns relating to future pregnancies which requires health‐care professionals to individualize the care they provide to parents after stillbirth. The complex needs of the mother and father highlight the importance of a multidimensional approach, including health‐ and social care professionals, especially in the area of follow‐up and future care.

## Competing Interests

None declared.

## Funding

No funding was sought or received for this study.
